# Machine learning models to predict the maximum severity of COVID-19 based on initial hospitalization record

**DOI:** 10.3389/fpubh.2022.1007205

**Published:** 2022-11-28

**Authors:** Suhyun Hwangbo, Yoonjung Kim, Chanhee Lee, Seungyeoun Lee, Bumjo Oh, Min Kyong Moon, Shin-Woo Kim, Taesung Park

**Affiliations:** ^1^Interdisciplinary Program in Bioinformatics, Seoul National University, Seoul, South Korea; ^2^Department of Genomic Medicine, Seoul National University Hospital, Seoul, South Korea; ^3^Department of Internal Medicine, School of Medicine, Kyungpook National University, Daegu, South Korea; ^4^Department of Mathematics and Statistics, Sejong University, Seoul, South Korea; ^5^Department of Family Medicine, Seoul Metropolitan Government Seoul National University Boramae Medical Center, Seoul, South Korea; ^6^Department of Internal Medicine, Seoul Metropolitan Government Seoul National University Boramae Medical Center, Seoul, South Korea; ^7^Department of Internal Medicine, Seoul National University College of Medicine, Seoul, South Korea; ^8^Department of Statistics, Seoul National University, Seoul, South Korea

**Keywords:** COVID-19, artificial intelligence, machine learning, severity, nomogram

## Abstract

**Background:**

As the worldwide spread of coronavirus disease 2019 (COVID-19) continues for a long time, early prediction of the maximum severity is required for effective treatment of each patient.

**Objective:**

This study aimed to develop predictive models for the maximum severity of hospitalized COVID-19 patients using artificial intelligence (AI)/machine learning (ML) algorithms.

**Methods:**

The medical records of 2,263 COVID-19 patients admitted to 10 hospitals in Daegu, Korea, from February 18, 2020, to May 19, 2020, were comprehensively reviewed. The maximum severity during hospitalization was divided into four groups according to the severity level: mild, moderate, severe, and critical. The patient's initial hospitalization records were used as predictors. The total dataset was randomly split into a training set and a testing set in a 2:1 ratio, taking into account the four maximum severity groups. Predictive models were developed using the training set and were evaluated using the testing set. Two approaches were performed: using four groups based on original severity levels groups (i.e., 4-group classification) and using two groups after regrouping the four severity level into two (i.e., binary classification). Three variable selection methods including randomForestSRC were performed. As AI/ML algorithms for 4-group classification, GUIDE and proportional odds model were used. For binary classification, we used five AI/ML algorithms, including deep neural network and GUIDE.

**Results:**

Of the four maximum severity groups, the moderate group had the highest percentage (1,115 patients; 49.5%). As factors contributing to exacerbation of maximum severity, there were 25 statistically significant predictors through simple analysis of linear trends. As a result of model development, the following three models based on binary classification showed high predictive performance: (1) Mild vs. Above Moderate, (2) Below Moderate vs. Above Severe, and (3) Below Severe vs. Critical. The performance of these three binary models was evaluated using AUC values 0.883, 0.879, and, 0.887, respectively. Based on results for each of the three predictive models, we developed web-based nomograms for clinical use (http://statgen.snu.ac.kr/software/nomogramDaeguCovid/).

**Conclusions:**

We successfully developed web-based nomograms predicting the maximum severity. These nomograms are expected to help plan an effective treatment for each patient in the clinical field.

## Introduction

The coronavirus disease 2019 (COVID-19) pandemic is a rapidly evolving global emergency that continues to strain healthcare systems (1). Vaccinations are currently being implemented worldwide, but the pandemic persists and it leads to increases in the demand for medical resources. The clinical course of COVID-19 patients is known to appear in various forms ranging from asymptomatic to critical. A large cohort study that included 44,672 patients with COVID-19 from China showed that most cases were classified as mild to moderate (81%). However, 14% were severe, and 5% were critical (2). Therefore, to date, studies on various clinical parameters have been conducted to develop predictive scores or algorithms to identify clinical courses in the early stage.

Age and underlying diseases are known factors associated with higher risks of increased severity or mortality in patients with COVID-19 (3–5). In other studies, clinical symptoms, and laboratory or radiologic findings were included in the factors predicting severity or mortality associated with COVID-19 (6–8). There are several studies considering all of these clinical factors. For example, a retrospective study conducted in China showed that old age, coronary heart disease condition, lymphopenia, elevated procalcitonin, and D-dimer were independently related to mortality (9). Another study conducted in Switzerland for predicting severe disease courses requiring ICU admission demonstrated that male sex, low hemoglobin, the elevation of inflammatory parameters [C-reactive protein (CRP) or leucocyte counts], hyperglycemia, and impaired renal function were the most predictive risk factors (10).

As pointed out by Kim et al., it is important to prioritize patients in need of intensive care to avoid unnecessary consumption of medical resources on mild patients (11). This importance was further emphasized as the sudden COVID-19 outbreak intensified the shortage of hospital beds, critical care equipment, and medical professionals (12). To efficiently manage limited medical resources, it is important to predict the clinical course of patients during hospitalization. It is expected to properly triage patients, monitor the clinical progress of the disease, and allocate proper resources including intensive care facilities or healthcare staff by predicting the maximum severity of clinical progress.

Various nomograms have been developed for predicting the probabilities of disease progression or COVID-19-related mortality using baseline characteristics of patients (13–15). Specifically, we previously developed nomograms that predict the triage for COVID-19 patients based on 5,601 Korean patients (15). Although a large number of patients were included at the time, there were some restrictions on data access and software availability imposed by the Korea Disease Control and Prevention Agency (KDCA). Only three traditional machine learning (ML) algorithms [i.e., logistic regression (LR) (16), random forest (RF) (17), and support vector machine (SVM) (18)] could be applicable. For laboratory data, only five blood cell-centric findings were available. In addition, as initial chest X-ray and inflammatory laboratory findings have been reviewed as factors for severity prediction (1, 19), we further collected these radiologic and laboratory findings to develop more accurate predictive models.

In this study, we developed early predictive models of the maximum severity after the diagnosis of COVID-19. In addition to the three ML algorithms used in our previous study, we were able to apply more sophisticated artificial intelligence (AI)/ML algorithms such as GUIDE (20) and deep neural network (DNN) (21) because newly collected data were used instead of the public data provided by KDCA. We also added initial chest X-ray infiltration and various additional laboratory findings including inflammatory index (i.e. CRP) and organ dysfunction markers [i.e., aspartate transaminase (AST), creatinine, lactate dehydrogenase (LDH)] as candidate predictors (22). Our studies provide evidence that AI/ML applied to clinical parameters are expected to enable the development of tools that can predict the maximum severity.

## Materials and methods

### Study design

This is a multicenter retrospective cohort study of polymerase chain reaction-confirmed COVID-19 patients admitted to 10 hospitals in Daegu, Korea (23). The cohort includes data from 2,263 patients followed from February 18, 2020, to May 19, 2020. The data records consist of demographic characteristics, physical measurement, vital signs, clinical findings (i.e., symptoms), co-morbidities, radiologic findings, and laboratory findings. For readmitted patients, first admission records were used.

A total of 46 variables were used in this study. Excluding an outcome variable (i.e., maximum severity during hospitalization), 45 variables were used as predictors. Records for 45 predictors with an average missing rate of 16% (IQR: 6–19%) were collected from each patient on the first day of admission. In this study, the original data was used as it is. This study was approved by the institutional review board of Kyungpook National University Hospital (KNUH 2020-03-044).

### Data preprocessing

Excluding those who died on the first day of admission, 2,254 of 2,263 patients were used in this study. To define the outcome, a disease severity variable was used. The disease severity was divided into four groups: mild, moderate, severe, and critical (23). The disease severity was systematically defined by reflecting the opinions of infectious disease specialists in the clinical field. Detailed definitions of the four groups are given in Table 1. Each patient's diagnosis places them among the four severity groups, depending on the patient's condition (i.e., body temperature, diagnosis of pneumonia, oxygen therapy) during hospitalization. The highest level of severity for a patient diagnosed during hospitalization was called maximum severity. This is to predict and prepare in advance for high-risk patients for conditions such as pneumonia, ICU admission, and death that occur suddenly during hospitalization.

**Table 1:** Four groups of disease severity.

**Disease severity**	**Description**
Mild	No pneumonia
Moderate	Diagnosis of pneumonia but not requiring oxygen therapy
Severe	Diagnosis of pneumonia and the need for oxygen therapy
Critical	Diagnosis of pneumonia, need for mechanical ventilation therapy or extracorporeal membrane oxygenation, or death

Of the 45 predictors, 2 demographic variables, 4 vital signs, and 12 laboratory findings were continuous variables. Note that the body temperature belonging to the four vital sign predictors is the body temperature measured only on the first day of admission. Based on the opinions of clinicians for practical use in the clinical field, an optimal cutoff was selected for dichotomizing each continuous predictor. To this end, the maximally selected rank statistics (24) were used. Thus, all predictors used in this study were discrete variables. For each predictor variable, the Cochran-Armitage Trend test (CA) (25) was performed to identify the predictor with a linear trend of the maximum severity. Specifically, a one-sided test was performed to identify predictor with increasing linear trend. That is, in the case of significant variables, the proportion increases as the severity of the disease increases in the group exceeding the cutoff. Bonferroni correction was applied to handle multiple testing problem (26). A *p*-value < 0.05 was considered significant.

### Multiple marker selection

**Figure 1:**
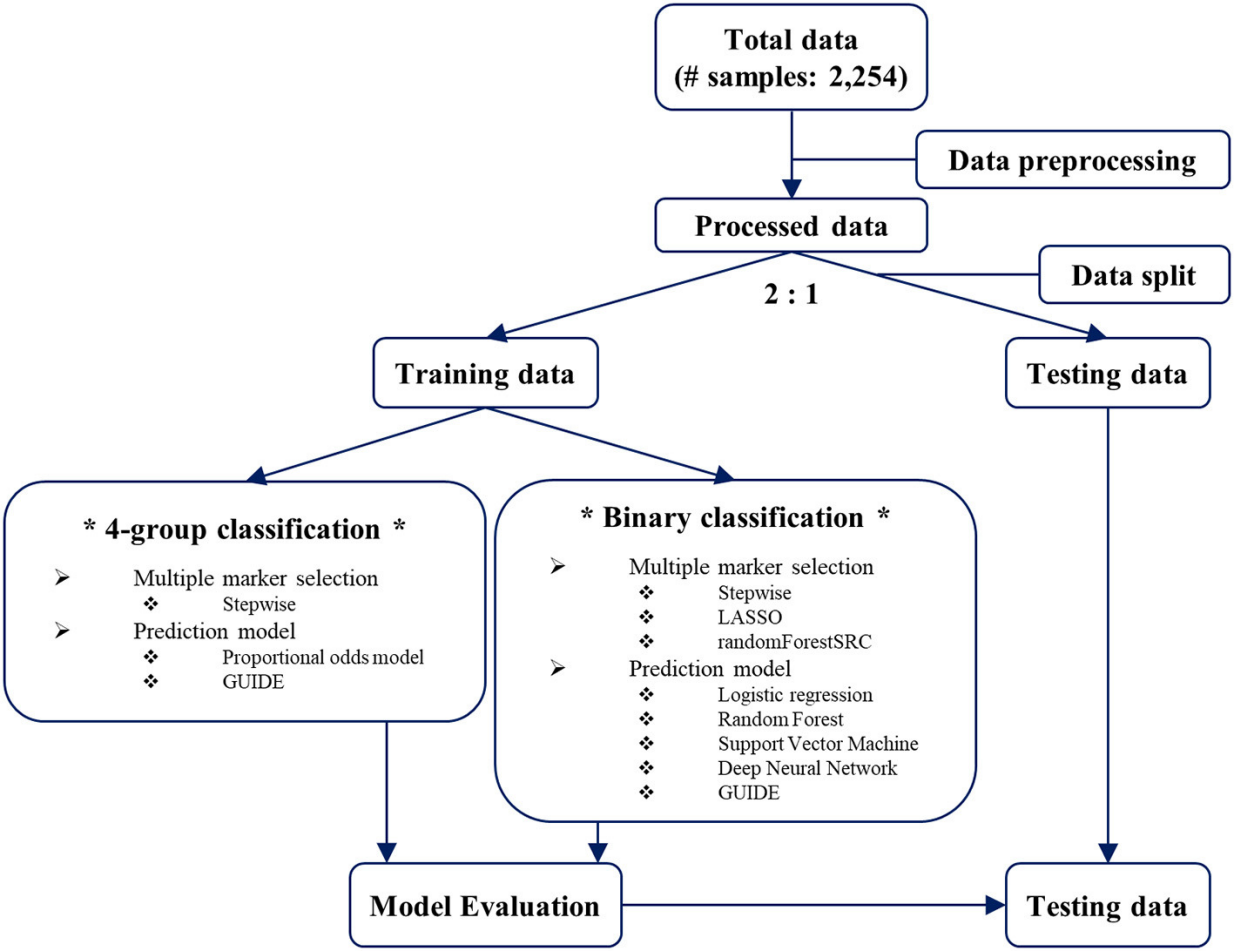
The overall workflow for predictive model development.

The overall workflow is shown in Figure 1. To avoid overfitting in the predictive model, we randomly split the total dataset into training and testing datasets in a 2:1 ratio, taking into account the maximum severity proportions of the four groups. While developing the predictive model for the maximum severity, two types of classification were considered: 4-group classification and binary classification. For the 4-group classification, we developed a predictive model using four maximum severity groups as they are. For multiple marker selection, Akaike information criterion (AIC)-based stepwise selection (27) was used.

For the binary classification, we combined the four maximum severity groups into two groups: (1) Mild vs. Above Moderate (2) Below Moderate vs. Above Severe (3) Below Severe vs. Critical. Above Moderate refers to a group that combines moderate, severe, and critical. Below Moderate refers to a group that combines mild and moderate. Above Severe refers to a group that combines severe and critical. Below Severe refers to a group that combines mild, moderate, and severe. For each outcome with a binary group, multiple predictive markers were selected using the area under the receiver operating characteristic curve (AUC)-based stepwise selection (28), the least absolute shrinkage and selection operator (LASSO) (29), and randomForestSRC (30). For randomforestSRC, we used both GINI index and AUC as a splitting rule. To compare the performance of LR-based predictors (stepwise and LASSO) and randomforestSRC given the same number of predictors, we matched the number of predictors with the average number (i.e., 5) of LR-based predictors when selecting the top ranked predictors in the randomforestSRC. For each binary outcome, the top five predictors were selected based on the variable importance result of randomforestSRC.

### Model development and evaluation

For the 4-group classification, the proportional odds model (31) and GUIDE were used (Figure 1). The proportional odds model is an extended logistic regression for the ordinal outcome as follows:


(1) $$\begin{array}{l} log[\mathrm{Pr}\left(Y\leq j|X\right)/\mathrm{Pr}\left(Y>j|X\right)]\;=\;\alpha_{j}-\beta^{T}X, \end{array}$$

where *Y* is the outcome with four ordinal categories (*j* = *mild, moderate, severe*), α_*j*_ is an intercept corresponding to the *j*^*th*^ category and β is a vector of coefficients. In the case of the proportional odds model, the proportionality assumption was confirmed through the likelihood ratio test, which compares the proportional odds model and the cumulative logit model (32). GUIDE is an improved decision tree-based method and develops a predictive model by selecting predictors based on the Chi-square test (20). As evaluation measures, precision, recall, and F1-score were calculated by comparing each category to the rest using a one-vs-all strategy. For each of these measures, two types of averages were calculated with considering the sample size and without considering the sample size. In addition, the accuracy was used by calculating the proportion of correctly classified observations in the confusion matrix.

For the binary classification, we developed three predictive models: (1) Mild vs. Above Moderate (2) Below Moderate vs. Above Severe (3) Below Severe vs. Critical. During the marker selection process, a 5-fold cross-validation (CV) was performed. We considered five AI/ ML algorithms: LR, RF, SVM, DNN, and GUIDE. For RF, SVM, and DNN, we tuned hyperparameters *via* 5-fold CV to identify optimal hyperparameters with the highest mean of AUCs. In addition to AUC, balanced accuracy, specificity, recall (i.e., sensitivity), precision, and F1-score were used as evaluation measures. A parsimonious model, a simple model with a high predictive ability for each outcome, was considered the final predictive model.

## Results

### Patient's characteristics

For the total of 2,254 COVID-19 patients, the maximum severity was classified into four groups. The four groups are mild (*n* = 548; 24.3%), moderate (*n* = 1,115; 49.5%), severe (*n* = 397; 17.6%), and critical (*n* = 194; 8.6%). Table 2 shows the clinical characteristics of 2,254 patients for the four groups. For age, the proportion of severe and critical groups over 60 years was very high compared to those under 60 years old (adj. *p*-value = 2.8E-98; CA test). In terms of sex, the maximum severity was more severe for men than women (adj. *p*-value = 0.0043; CA test). Body mass index (BMI) and two vital signs (i.e., body temperature, and respiration rate) were statistically significant predictors showing a linear trend with the maximum severity. Among the other predictors, three initial clinical findings were fatigue, shortness of breath, altered consciousness, 8 comorbidities [i.e., diabetes, heart failure, hypertension, chronic cardiac disease (CCD), chronic kidney disease, cancer, chronic neurological disorder (CND), dementia], chest X-ray infiltration, and 8 laboratory findings [i.e., CRP, Procalcitonin, AST, alanine transaminase (ALT), Creatine kinase MB, LDH, Creatinine, prothrombin time international normalized ratio (PT-INR)] showed the linear trend with the maximum severity (Table 2).

**Table 2:** Patient's characteristics for each group of the maximum severity.

**Characteristics**	**Mild** **[*n* = 548; 24.3%]**	**Moderate** **[*n* = 1,115; 49.5%]**	**Severe** **[*n* = 397; 17.6%]**	**Critical** **[*n* = 194; 8.6%]**	*P*-value^*^	**adj. *P*-value ^¥^**
**Demographics**						
Age [>60 year]	95 (17%)	433 (39%)	290 (73%)	171 (88%)	6.2E-100	2.8E-98
Sex [Male]	205 (37%)	343 (31%)	147 (37%)	108 (56%)	9.6E-05	0.0043
**Physical measurement**						
BMI [>25 $\frac{kg}{m^{2}}$]	103 (25%)	194 (27%)	82 (33%)	46 (40%)	2.6E-04	0.0117
**Initial vital signs**						
Body temperature [>37.4°C]	0 (0%)	249 (22%)	110 (28%)	62 (32%)	2.0E-32	9.0E-31
SBP [>140 mmHg]	166 (31%)	376 (35%)	141 (36%)	74 (39%)	0.0218	0.9810
Heart rate [>100 $\frac{beats}{\min}$]	92 (17%)	186 (17%)	69 (17%)	48 (25%)	0.0270	1
Respiration rate [>20 $\frac{breaths}{\min}$]	45 (8%)	105 (10%)	90 (23%)	93 (49%)	6.2E-41	2.8E-39
**Clinical findings [Yes]**						
Cough	114 (34%)	491 (50%)	192 (51%)	66 (35%)	0.1543	1
Sputum	106 (32%)	362 (37%)	139 (37%)	64 (34%)	0.2413	1
Sore throat	44 (13%)	141 (15%)	43 (12%)	10 (5%)	0.9964	1
Runny nose	48 (15%)	100 (10%)	34 (9%)	10 (5%)	0.9993	1
Muscle aches	47 (15%)	233 (24%)	87 (25%)	21 (12%)	0.5682	1
Fatigue	5 (2%)	31 (3%)	21 (6%)	16 (9%)	2.7E-06	1.2E-04
Shortness of breath	26 (8%)	140 (14%)	130 (36%)	100 (54%)	1.1E-47	4.9E-46
Headache	51 (15%)	254 (26%)	66 (18%)	12 (7%)	0.9982	1
Altered consciousness	0 (0%)	3 (0%)	4 (1%)	15 (8%)	9.3E-14	4.2E-12
Vomiting	10 (3%)	71 (7%)	33 (9%)	11 (6%)	0.0300	1
Diarrhea	38 (12%)	149 (15%)	51 (14%)	16 (9%)	0.8123	1
**Comorbidities & past history [Yes]**						
Diabetes	43 (8%)	152 (14%)	105 (27%)	76 (41%)	3.8E-32	1.7E-30
Heart failure	3 (1%)	11 (1%)	17 (5%)	11 (6%)	1.7E-09	7.6E-08
Hypertension	74 (14%)	270 (24%)	184 (47%)	113 (59%)	2.1E-49	9.4E-48
Chronic cardiac disease	7 (1%)	43 (4%)	40 (11%)	20 (11%)	1.9E-13	8.5E-12
Asthma	11 (2%)	33 (3%)	12 (3%)	9 (6%)	0.0139	0.6255
COPD	5 (1%)	11 (1%)	10 (3%)	5 (3%)	0.0030	0.1350
Chronic kidney disease	4 (1%)	6 (1%)	13 (4%)	14 (9%)	9.6E-12	4.3E-10
Cancer	10 (2%)	39 (4%)	18 (5%)	20 (12%)	3.4E-08	1.5E-06
Chronic liver disease	5 (1%)	19 (2%)	11 (3%)	4 (2%)	0.0173	0.7785
Chronic neurological disorder	1 (0%)	4 (0%)	2 (1%)	8 (5%)	6.0E-07	2.7E-05
Chronic hematologic disease	3 (1%)	7 (1%)	5 (2%)	4 (2%)	0.0413	1
RDAD	2 (1%)	7 (1%)	3 (1%)	3 (2%)	0.1020	1
Dementia	4 (1%)	54 (6%)	69 (21%)	48 (29%)	6.8E-33	3.1E-31
Smoking	46 (10%)	58 (7%)	30 (9%)	21 (12%)	0.1777	1
**Radiologic finding [Yes]**						
Chest X-ray infiltration	0 (0%)	565 (51%)	263 (67%)	155 (82%)	1.7E-116	7.6E-115
**Laboratory tests**						
CRP [>3 mg/dL]	1 (1%)	72 (14%)	140 (61%)	122 (91%)	1.1E-92	4.9E-91
Procalcitonin [>0.25 mg/dL]	2 (2%)	8 (2%)	27 (12%)	42 (37%)	7.2E-26	3.2E-24
AST [>40 U/L]	24 (7%)	113 (11%)	121 (31%)	107 (55%)	3.9E-54	1.7E-52
ALT [>40 U/L]	36 (11%)	121 (12%)	83 (22%)	39 (20%)	2.3E-06	1.0E-04
Creatine kinase MB [>5 ng/mL]	2 (1%)	7 (2%)	13 (6%)	23 (17%)	4.8E-11	2.2E-09
LDH [>400 ng/mL]	127 (41%)	514 (57%)	224 (68%)	113 (77%)	2.9E-17	1.3E-15
Creatinine [>1.3 mg/dL]	5 (2%)	26 (3%)	41 (11%)	55 (28%)	2.8E-36	1.3E-34
Hemoglobin [>10 g/dL]	323 (98%)	954 (96%)	348 (90%)	158 (81%)	1	1
Lymphocyte [>50%]	19 (6%)	48 (5%)	6 (2%)	2 (1%)	0.9999	1
PT-INR [>1.5]	0 (0%)	7 (1%)	4 (2%)	11 (7%)	2.8E-06	1.3E-04
Platelet [$>150\frac{10^{3}}{uL}$]	317 (95%)	857 (86%)	293 (76%)	128 (66%)	1	1
White blood cell [$>4\frac{10^{3}}{uL}$]	292 (88%)	768 (77%)	307 (80%)	174 (90%)	0.3503	1

For each binary predictor, the number in the square bracket indicates the cutoff for binarization. The percentage of each group represents the proportion of the number of groups that meet the > cutoff excluding missing values.

* The p-value was calculated using the Cochran-Armitage Trend test.

¥ The adjusted p-value was calculated using the Bonferroni correction method. BMI, Body mass index; SBP, systolic blood pressure; COPD, Chronic obstructive pulmonary disease; RDAD, Rheumatic disorder/Autoimmune disease; CRP, C-reactive protein; AST, Aspartate transaminase; ALT, Alanine transaminase; LDH, Lactate dehydrogenase; PT-INR, Prothrombin time international normalized ratio.

### Predictive models for 4-group classification

First, we developed predictive models using the four ordinal groups which represent triage COVID-19 patients more informatively. To select multiple markers associated with the maximum severity, we used the proportional odds model and GUIDE model. As a result of AIC-based stepwise selection, eight predictors were selected including age, SBP, cough, sore throat, shortness of breath, hypertension, ALT, and lymphocyte. Based on these eight predictors, the proportional odds assumption was held (*p*-value = 0.9980). Thus, we developed a predictive model using the proportional odds model and evaluated its performance on the testing data. In the case of the proportional odds model when evaluating the performance, the probability of being a specific category *j* was calculated by using the difference between the cumulative probability corresponding to *j* and *j*−1 [i.e., *P*(*Y* = *j*) = *P*(*Y* ≤ *j*)−*P*(*Y* ≤ *j*−1)]. Each sample of the testing data is classified into the group with the highest probability. The evaluation results of the proportional odds model are shown in Table 3. The accuracy and the weighted averages of precision, recall, and F1-scores for the proportional odds model was 0.524, 0.407, 0.499, and 0.408, respectively. For the GUIDE model, six predictors were selected as follows: CRP, respiration rate, age, headache, cough, and PT-INR. The prediction results of the GUIDE model were also low (Table 3). In both the proportional odds model and GUIDE, it seems that most groups were predicted to be the moderate group with the highest proportion of the four severity groups.

**Table 3:** Predictive models and performance for 4-group classification in testing data.

**Model**	**Accuracy**	**Weighted average**			**Macro average**		
		**Precision**	**Recall**	**F1-score**	**Precision**	**Recall**	**F1-score**
Proportional odds model	0.524	0.407	0.499	0.408	0.329	0.296	0.270
GUIDE	0.564	0.553	0.562	0.542	0.513	0.433	0.449

F1-score is defined as the harmonic mean of precision and recall.

### Predictive models for binary classification

By extending the approach of our previous study, we developed predictive models based on more sophisticated AI/ML algorithms using a variety of predictors such as radiologic and laboratory findings reported to be associated with severity. We combined the four outcome groups into two groups [i.e., (1) Mild vs. Above Moderate, (2) Below Moderate vs. Above Severe, and (3) Below Severe vs. Critical]. For each of the three binary outcomes, the variable selection was performed using AUC-based stepwise, LASSO, and randomForestSRC methods. For each outcome, predictive models were developed using selected variables based on five AI/ML algorithms: LR, RF, SVM, DNN, and GUIDE. Table 4 shows the model and performance results. For each binary outcome, a parsimonious model with the best predictive performance using fewer predictors was chosen as the final model.

**Table 4:** Predictive models and performance for each binary outcome.

**Binary outcome**	**Num^¥^**	**Variable selection method**	**AI/ML**	**Training**						**Testing**					
				**AUC**	**Acc**.	**Spe**.	**Prec**.	**Rec**.	**F1**	**AUC**	**Acc**.	**Spe**.	**Prec**.	**Rec**.	**F1**
Mild vs. Above Moderate	8	Stepwise	LR	0.918	0.867	0.996	0.998	0.739	0.849	0.894	0.847	0.985	0.992	0.710	0.827
			RF	0.915	0.857	0.941	0.973	0.774	0.862	0.879	0.825	0.901	0.950	0.749	0.838
			SVM	0.911	0.867	0.992	0.996	0.742	0.850	0.881	0.843	0.969	0.983	0.716	0.829
			DNN	0.916	0.865	0.974	0.988	0.755	0.856	0.890	0.838	0.937	0.967	0.738	0.837
	4	LASSO	LR	0.895	0.845	1.000	1	0.691	0.817	0.871	0.844	1.000	1	0.689	0.816
			RF	0.863	0.815	0.923	0.977	0.706	0.820	0.836	0.807	0.925	0.978	0.689	0.808
			SVM	0.896	0.845	1.000	1	0.691	0.817	0.871	0.845	1.000	1	0.689	0.816
			DNN	0.894	0.832	0.961	0.988	0.703	0.822	0.870	0.807	0.864	0.964	0.749	0.843
	5	RandomForestSRC (Split rule: GINI)	LR	0.883	0.850	1	1	0.700	0.823	0.861	0.853	1	1	0.705	0.827
			RF	0.822	0.818	0.835	0.970	0.801	0.877	0.741	0.757	0.711	0.944	0.804	0.868
			SVM	0.880	0.841	0.988	0.997	0.694	0.818	0.863	0.847	1	1	0.694	0.819
			DNN	0.875	0.830	0.945	0.989	0.715	0.828	0.867	0.816	0.884	0.978	0.747	0.845
	5	RandomForestSRC (Split rule: AUC)	LR	0.845	0.800	0.981	0.993	0.620	0.763	0.823	0.788	0.980	0.993	0.595	0.744
			RF	0.855	0.803	0.977	0.992	0.630	0.771	0.831	0.791	0.980	0.993	0.601	0.749
			SVM	0.849	0.796	1	1	0.591	0.743	0.827	0.786	1	1	0.572	0.728
			DNN	0.852	0.802	0.980	0.993	0.623	0.766	0.828	0.788	0.984	0.995	0.593	0.743
	3^*^	GUIDE		0.891	0.835	0.841	0.942	0.830	0.882	0.882	0.811	0.797	0.927	0.826	0.874
Below moderate vs. above severe	8	Stepwise	LR	0.915	0.838	0.851	0.729	0.825	0.774	0.878	0.825	0.824	0.653	0.825	0.729
			RF	0.919	0.849	0.905	0.802	0.794	0.798	0.875	0.821	0.804	0.632	0.837	0.720
			SVM	0.912	0.839	0.867	0.747	0.810	0.777	0.877	0.816	0.794	0.621	0.838	0.713
			DNN	0.915	0.831	0.865	0.741	0.797	0.768	0.876	0.810	0.839	0.661	0.780	0.715
	5^*^	LASSO	LR	0.899	0.827	0.869	0.759	0.786	0.772	0.879	0.799	0.864	0.726	0.733	0.730
			RF	0.902	0.830	0.887	0.783	0.772	0.778	0.873	0.806	0.841	0.704	0.771	0.736
			SVM	0.901	0.830	0.887	0.782	0.772	0.777	0.877	0.802	0.822	0.683	0.781	0.729
			DNN	0.899	0.825	0.889	0.783	0.760	0.771	0.873	0.797	0.866	0.727	0.728	0.728
	5	RandomForestSRC (Split rule: GINI)	LR	0.892	0.828	0.885	0.778	0.771	0.775	0.856	0.797	0.794	0.656	0.800	0.721
			RF	0.892	0.828	0.889	0.784	0.767	0.775	0.859	0.801	0.733	0.733	0.869	0.733
			SVM	0.890	0.828	0.889	0.784	0.767	0.775	0.859	0.801	0.869	0.733	0.733	0.733
			DNN	0.890	0.829	0.868	0.761	0.789	0.773	0.856	0.796	0.848	0.707	0.745	0.725
	5	RandomForestSRC (Split rule: AUC)	LR	0.890	0.823	0.896	0.791	0.749	0.770	0.859	0.794	0.804	0.667	0.785	0.721
			RF	0.895	0.828	0.831	0.719	0.825	0.768	0.855	0.797	0.790	0.656	0.804	0.723
			SVM	0.888	0.822	0.899	0.794	0.744	0.768	0.862	0.794	0.804	0.667	0.785	0.721
			DNN	0.888	0.820	0.863	0.754	0.778	0.763	0.859	0.786	0.839	0.697	0.733	0.713
	4	GUIDE		0.793	0.738	0.698	0.477	0.778	0.592	0.776	0.716	0.650	0.444	0.782	0.566
Below severe vs. critical	6^*^	Stepwise	LR	0.928	0.876	0.855	0.496	0.896	0.639	0.887	0.848	0.774	0.304	0.923	0.457
			RF	0.928	0.878	0.820	0.453	0.935	0.610	0.878	0.832	0.856	0.375	0.808	0.512
			SVM	0.928	0.870	0.805	0.433	0.935	0.592	0.885	0.838	0.753	0.286	0.923	0.436
			DNN	0.922	0.857	0.865	0.500	0.848	0.629	0.886	0.822	0.829	0.338	0.815	0.478
	1	LASSO	LR	0.845	0.846	0.775	0.394	0.916	0.551	0.819	0.819	0.740	0.307	0.897	0.457
			RF	0.845	0.845	0.775	0.394	0.916	0.551	0.819	0.819	0.740	0.307	0.897	0.457
			SVM	0.845	0.846	0.775	0.394	0.916	0.551	0.819	0.819	0.740	0.307	0.897	0.457
			DNN	0.846	0.570	0.356	0.163	0.784	0.270	0.820	0.566	0.347	0.134	0.785	0.228
	5	RandomForestSRC (Split rule: GINI)	LR	0.909	0.845	0.769	0.389	0.921	0.547	0.858	0.820	0.754	0.307	0.886	0.456
			RF	0.901	0.827	0.823	0.428	0.831	0.565	0.826	0.779	0.644	0.241	0.914	0.380
			SVM	0.902	0.825	0.696	0.333	0.955	0.494	0.843	0.790	0.665	0.252	0.914	0.395
			DNN	0.911	0.842	0.788	0.405	0.896	0.556	0.857	0.805	0.759	0.304	0.851	0.447
	5	RandomForestSRC (Split rule: AUC)	LR	0.916	0.846	0.877	0.524	0.815	0.638	0.868	0.819	0.715	0.305	0.923	0.459
			RF	0.920	0.847	0.879	0.528	0.815	0.641	0.856	0.819	0.715	0.305	0.923	0.459
			SVM	0.913	0.842	0.857	0.490	0.826	0.615	0.873	0.816	0.708	0.300	0.923	0.453
			DNN	0.913	0.845	0.851	0.495	0.840	0.619	0.865	0.814	0.710	0.301	0.918	0.453
	3	GUIDE		0.805	0.788	0.902	0.393	0.674	0.496	0.723	0.712	0.885	0.307	0.538	0.391

¥ The number of variables selected by the variable selection method.

* The final model used to develop the nomogram for each binary outcome. F1 score is defined as the harmonic mean of precision (Prec.) and recall (Rec.). Specificity, recall (Rec.; sensitivity), precision (Prec.), and F1-score at the threshold corresponding to the maximum balanced accuracy (Acc.) are displayed. For the Mild vs. Above Moderate model, the final model includes chest X-ray infiltration, body temperature, and age. For Below Moderate vs. Above Severe model, the final model includes age, shortness of breath, chest X-ray infiltration, CRP, and AST. For Below Severe vs. Critical model, the final model includes CRP, respiration rate, chronic kidney disease, AST, age, and diabetes. Acc, Accuracy; Spe., Specificity; Prec., Precision; Rec., Recall.

**Figure 2:**
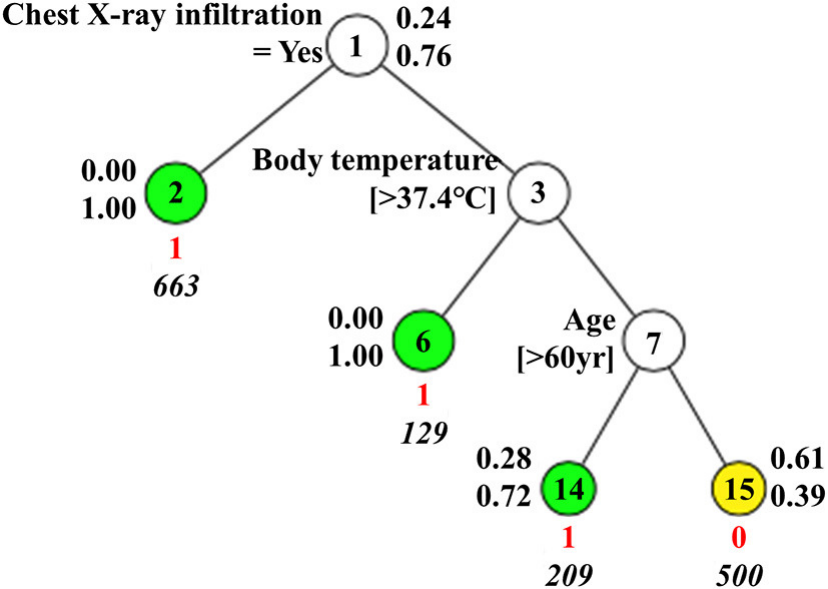
GUIDE model for Mild vs. Above Moderate.

For (1) Mild vs. Above Moderate model, three predictors including chest X-ray infiltration, body temperature, and age were finally selected for the final model. The final model showed good performance with an AUC of 0.882, balanced accuracy of 0.811, and F1-score of 0.874 for GUIDE. In particular, the predictive performance of the GUIDE for binary classification is much better than a model for 4-group classification, when converting the 4 × 4 confusion matrix of 4-group classification to a 2 × 2 version (33). Figure 2 shows the results of GUIDE using training data. The number between 1 and 15 of each node represents the label of the node. At each split, an observation goes to the left branch if and only if the condition is satisfied. The predicted class (in red) and sample size (in italics) are printed below the terminal node. Terminal nodes with classes predicted to be Above Moderate = 1 are shown in green, and classes predicted to be Mild = 0 are shown in yellow. Sample proportions by class for Mild = 0 and Above Moderate = 1 are displayed next to the node. From the tree, the importance of the variables can be inferred in the order of chest X-ray infiltration, body temperature, and age. In that an observation moves to the left branch when the condition is met, all predictors can be inferred to have positive effects on the Above Moderate group.

For (2) Below Moderate vs. Above Severe model, 5 predictors were finally selected as the final model: age, shortness of breath, chest X-ray infiltration, CRP, and AST. Based on LR with the highest performance, all predictors showed positive effects on the Above Severe group (Table 5). When ranked based on statistical significance, CRP had the highest, followed by shortness of breath. A positive association between CRP level and the severity of COVID-19 has been reported (34). In addition to CRP, age, shortness of breath, and chest X-ray infiltration were also well-known factors predicting the severity of COVID-19 (15, 35). The model showed predictive performance with the highest performance with AUC = 0.879 (balanced accuracy = 0.799, F1-score = 0.730) for LR.

**Table 5:** Fitted results of the logistic regression model for the Below Moderate vs. Above Severe model and Below Severe vs. Critical model.

**(A) Binary outcome: Below moderate vs. above severe**			
	**Estimate**	**Std. Error**	**Pr(>|z|)**
(Intercept)	−3.197	0.270	2.4E-32
Age [>60 year]	1.204	0.253	1.9E-06
Shortness of breath [Yes]	1.431	0.261	4.4E-08
Chest X-ray infiltration [Yes]	0.540	0.257	0.035
CRP [>3 mg/dL]	2.270	0.251	1.3E-19
AST [>40U/L]	1.144	0.298	1.2E-04
**(B) Binary outcome: Below severe vs. critical**			
(Intercept)	−5.605	0.575	1.8E-22
CRP [>3 mg/dL]	2.688	0.455	3.4E-09
Respiration rate [>20 breaths /min]	1.420	0.333	1.9E-05
Chronic kidney disease [Yes]	2.660	0.809	0.001
AST [>40U/L]	1.384	0.342	5.2E-05
Age [>60 year]	0.801	0.434	0.065
Diabetes [Yes]	1.085	0.346	0.002

**Figure 3:**
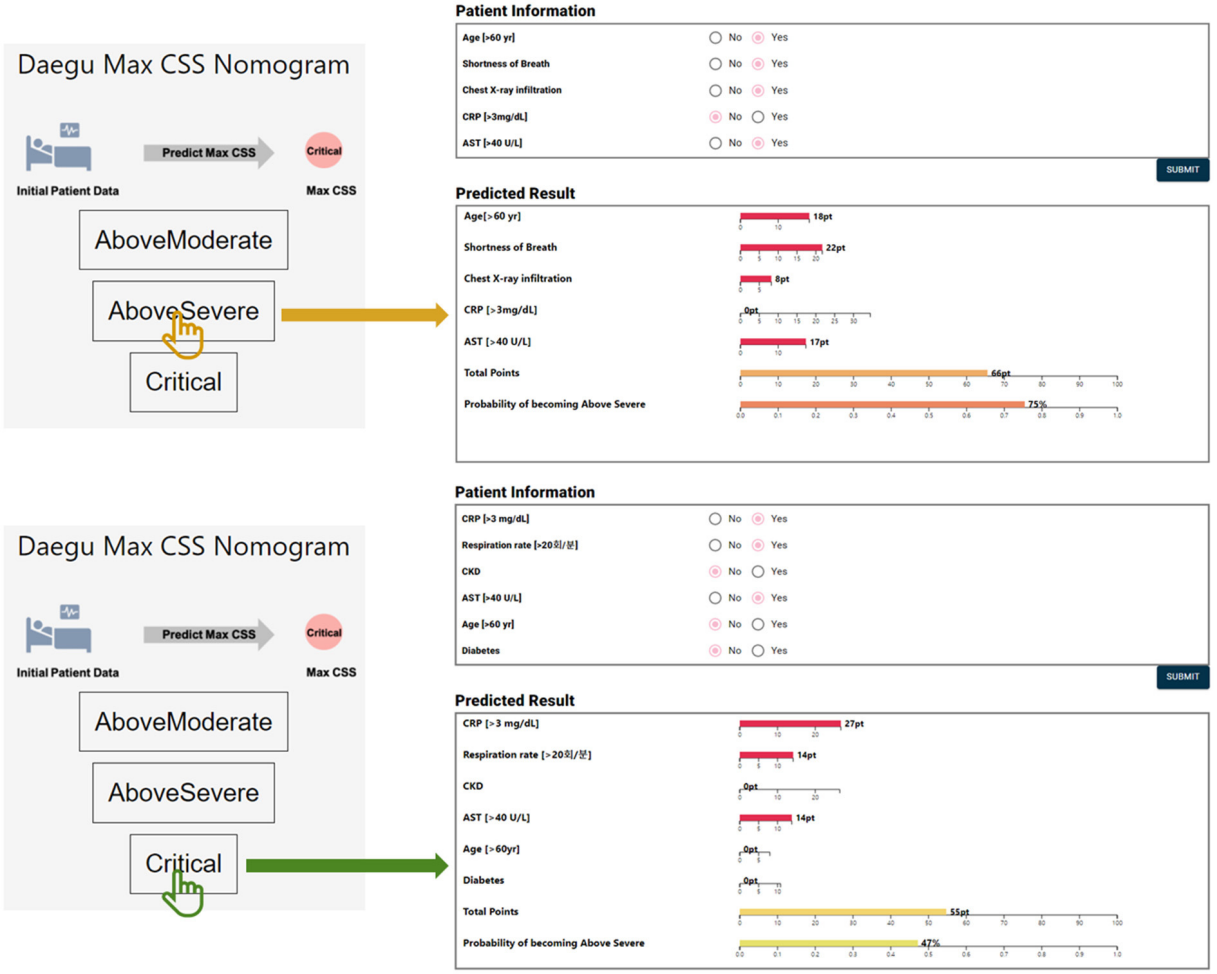
An example of the nomogram for each outcome.

For (3) Below Severe vs. Critical model, 6 predictors were finally selected as the final model: CRP, respiration rate, chronic kidney disease, AST, age, and diabetes. As with the Below Moderate vs. Above Severe model, based on LR with the highest AUC value, all predictors showed positive effects on the Critical group (Table 5). When ranked based on statistical significance, CRP ranked highest. CRP has also been studied as a factor to predict the need for mechanical ventilation (36). In addition to CRP, respiration rate and age have been reported as predictors for an increased risk of mechanical ventilation (37). Chronic kidney disease and diabetes were also risk factors contributing to death in hospitalized COVID-19 patients (38, 39). The model had the highest AUC value of 0.887 and the highest balanced accuracy of 0.848 for LR. For these three predictive models, we developed nomograms to predict the maximum severity of each COVID-19 patient using the coefficients of the LR model (Figure 3). The nomogram is available at http://statgen.snu.ac.kr/software/nomogramDaeguCovid/ and is expected to help plan effective treatment for each patient in a clinical setting.

## Discussion

As the COVID-19 pandemic continues for a long time, the importance of proper preparation and distribution of medical resources at an early stage is growing. Early prediction of the high-risk group for severe COVID-19 pneumonia is important because it can reduce mortality by providing timely treatment to critically ill patients such as the elderly (40, 41). For early prediction, this study successfully developed AI/ML-based models that predict the maximum severity of COVID-19 patients during hospitalization. Of the two approaches used in this study, the binary classification approach performed much better than the 4-group classification approach. Based on the binary classification results with higher performance, we developed web-based nomograms useful for clinical practice as follows: (1) Mild vs. Above Moderate, (2) Below Moderate vs. Above Severe, and (3) Below Severe vs. Critical. The Mild vs. Above Moderate model showed the predictive performance of AUC = 0.882 using only three clinicopathologic predictors (i.e., chest X-ray infiltration, body temperature, and age). This implies that three predictors without laboratory findings are sufficient to predict the Mild vs. Above Moderate model. Conversely, models predicting (2) Above Severe or (3) Critical groups required laboratory findings such as CRP and AST. For these two models, the predictive performance was further compared to the best model when predictors were selected without laboratory findings. For the Below Moderate vs. Above Severe, the final model used to develop the nomogram showed higher predictive performance (AUC range: 0.873–0.879; 5 predictors) than the best model (AUC range: 0.809–0.825; 8 predictors which include age, chest X-ray, body temperature, smoking, CND, respiration rate, hypertension, and sex) when clinicopathological predictors were selected without laboratory findings. Here, the 8 predictors based only on clinicopathological variables are not the same as the 8 predictors (CRP, AST, age, shortness of breath, dementia, diabetes, creatinine, and chest X-ray) selected by the stepwise method in Table 4. The Below Severe vs. Critical model (AUC range: 0.878–0.887; 6 predictors) used to develop the nomogram also outperformed the best model (AUC range: 0.719–0.831; 8 predictors which include age, respiration rate, smoking, chest X-ray, diabetes, body temperature, sex, and CCD) when only clinicopathological predictors were selected.

Most of the predictors used to develop the nomogram were found to be consistent with previously reported results in the literature. Age, the common predictor of the three models used in the nomogram, is known to be a major risk factor for clinical severity (1). Chest X-ray infiltration was previously reported as a predictor of detecting the moderate and severe groups with an accuracy of 0.86 or better (42). CRP, which serves as an early marker of inflammation, has also been reported as an early predictor of COVID-19 severity (34, 43). AST, which acts as a sign of liver damage, has also been reported as one of the important predictors for predicting severity (44). It is well-known that shortness of breath is prevalent in severe patients (45). Respiration rate, chronic kidney disease, and diabetes were reported risk factors for mechanical ventilation or death corresponding to the critical group (37–39).

Our study showed similar results in a large cohort retrospective study conducted in the United States. The previous study used 64 input variables, including vital signs, various laboratory findings, and comorbidities. As the previous study found that age, male sex, and liver disease were associated with higher clinical severity, the Below Severe vs. Critical model in this study had a high predictive performance with clinical parameters including age, male sex, and elevated AST relating to liver diseases. In the previous study, ferritin and d-dimer were used as input variables, but in our study, cytokine storm syndrome-related these blood tests occurring in severe COVID-19 were not included. However, a high predictive model was presented without using these laboratory findings, and through this, convenience in predicting the disease severity in clinical situations can be expected. Our study demonstrated that the predictive model has the potential to predict the maximum disease severity of patients with COVID-19 with high accuracy and to help healthcare systems in planning for surge medical capacity for COVID-19, especially in a situation where medical resources are limited.

Compared to our previous study (15), the main characteristics of this study are as follows. Firstly, in this study, the maximum severity defined based on the opinions of infectious disease specialists is expected to arouse sympathy from other clinicians in the clinical field. As a result, this model may be useful in clinical practice and the design of further clinical studies. Secondly, various AI/ML algorithms including DNN and GUIDE were used as comparison methods. The predictive performance based on various AI/ML algorithms suggests that the logistic model used to develop the nomogram outperforms other comparative models. Lastly, the results of this study suggest that various laboratory findings such as CRP and AST contribute to the higher predictive performance with a smaller number of predictors.

Most of the existing methods have focused on classifying two groups, such as mild and severe patients (46). However, a predictive model based on the 4-group classification allows for ease of diagnosis for four groups of COVID-19 patients without the need for three predictive models. In addition, for more accurate classification, it is necessary to develop a predictive model using the ordinal information of four groups (47). Although the predictive performance for the four groups was not good because most of the four groups were predicted as the moderate group with the highest proportion, the results are expected to be useful for designing future analysis plans such as an approach reflecting weights depending on the proportion of the outcome.

However, this study has some limitations. Firstly, we could not evaluate the impact of COVID-19 treatment, new COVID-19 variants, and vaccination status on the clinical severity course because this study was conducted in early COVID-19 pandemic patient groups. Secondly, full therapeutic options were not available such as remdesivir, tocilizumab (anti-IL-6 receptor monoclonal antibody), baricitinib (janus kinase inhibitor), and anti-SARS-CoV-2 monoclonal antibody. Thirdly, laboratory findings related to cytokine storm syndrome occurring in severe COVID-19 such as ferritin, interleukin 6 (IL-6), and d-dimer were not included. Ferritin (macrophage activation indicator) and IL-6 (T lymphocyte activation) are known to suspect cytokine storm syndrome in severe COVID-19 exacerbation (48). If laboratory test results related to these cytokine systems were included, it could help to create a model that can better predict severe or critical disease severity. We are continually updating data from 10 hospitals in Daegu, and collecting new data at SMG-SNU Boramae Medical Center in Seoul. Based on these updated data, we are conducting new systematic analyzes including variant information and therapeutic options. The new results will be reported in a separate paper in the new future. Lastly, although our study included only Korean, further research on different races could help to predict clinical disease severity more accurately. However, despite these limitations, this study is a meaningful study in its way to determine the maximum severity of the patient only from the initial condition in the absence of variant information or treatment for COVID-19.

In conclusion, three predictive models were developed to predict the maximum severity during hospitalization based on the initial hospitalization records. The five AI/ML algorithms including DNN and GUIDE were used for model development. Each of the three predictive models showed excellent predictive performance using a few predictors. Representatively, the Mild vs. Above Moderate model showed the predictive performance of 0.882 for AUC using three clinicopathologic predictors. Based on these three predictive models, we successfully developed web-based nomograms useful in the clinical field. These nomograms are expected to help plan effective and timely treatment for each patient.

## Data availability statement

The datasets generated for this study are available on request to the corresponding author.

## Ethics statement

The studies involving human participants were reviewed and approved by the Institutional Review Board of Kyungpook National University Hospital (KNUH 2020-03-044). The patients/participants provided their written informed consent to participate in this study.

## Author contributions

SH and TP led the overall study and conceived the model. YK and S-WK contributed to the data collection. SH and CL contributed to the data analysis. CL developed the nomogram. SH, SL, BO, MM, S-WK, and TP contributed to data interpretation. SH and YK wrote the manuscript. TP and S-WK supervised the project. All authors read, edited, and approved the final manuscript.

## Funding

This research was supported by the Bio and Medical Technology Development Program of the National Research Foundation (NRF) funded by the Korean government (MSIT) (No. 2021M3E5E3081425).

## Conflict of interest

The authors declare that the research was conducted in the absence of any commercial or financial relationships that could be construed as a potential conflict of interest.

## Publisher's note

All claims expressed in this article are solely those of the authors and do not necessarily represent those of their affiliated organizations, or those of the publisher, the editors and the reviewers. Any product that may be evaluated in this article, or claim that may be made by its manufacturer, is not guaranteed or endorsed by the publisher.
